# Long Distance From Microvessel to Cancer Cell Predicts Poor Prognosis in Non-Small Cell Lung Cancer Patients

**DOI:** 10.3389/fonc.2021.632352

**Published:** 2021-06-11

**Authors:** Haiying Ding, Jiao Sun, Yu Song, Wenxiu Xin, Junfeng Zhu, Like Zhong, Yinbo Chen, Yiwen Zhang, Yinghui Tong, Luo Fang

**Affiliations:** ^1^ Department of Pharmacy, The Cancer Hospital of the University of Chinese Academy of Sciences (Zhejiang Cancer Hospital), Institute of Basic Medicine and Cancer (IBMC), Chinese Academy of Sciences, Hangzhou, China; ^2^ Department of Colorectal Cancer, The Cancer Hospital of the University of Chinese Academy of Sciences (Zhejiang Cancer Hospital), Institute of Basic Medicine and Cancer (IBMC), Chinese Academy of Sciences, Hangzhou, China; ^3^ Department of Pharmacy, Zhejiang Provincial People’s Hospital, People’s Hospital of Hangzhou Medical College, Hangzhou, China

**Keywords:** non-small cell lung cancer, prognosis marker, distance from microvessel to cancer cell, progression free survival, overall survival

## Abstract

**Background:**

Blood supply, which is crucial for nutrition and drug delivery, was determined by microvessel density as well as the diffusion distance between vessels and cancer cells. Therefore, we evaluated the distance from microvessels to cancer cells (D_mvcc_) and its role in the prognosis of non-small cell lung cancer (NSCLC) patients.

**Methods:**

Patients with primary NSCLC were retrospectively analyzed. The tumor samples were immunochemically stained with CD31 to visualize the microvessels. The D_mvcc_ was defined as the mean distance from each microvessel to its nearest cancer cell in the “hot-spot” of an individual patient. The patients were stratified into short- and long-distance groups using five strategies, including dichotomy by the median value, optimal cutoff, trichotomy, quartation and per-10 µm increase. The correlation between the D_mvcc_ and survival was evaluated by using univariate and multivariate analyses with various D_mvcc_ strategies.

**Results:**

In total, 100 patients were analyzed. The median value of D_mvcc_ was 13.1 μm (ranged, 1.6 to 269.7 μm; mean value, 24.4 ± 33.5 μm). The optimal cutoff value of D_mvcc_ for predicting survival outcome was 20 μm. D_mvcc_ was significantly related to overall survival (OS) with all the five categories (p = 0.001–0.000004) and progression-free survival (PFS) categorized by optimal cutoff value (p = 0.024), trichotomy (p = 0.041) and per-10 µm increase (p = 0.040) after adjusting for other factors. Patients with longer D_mvcc_ (≥20 μm) were observed to have poor survival outcomes (OS: HR = 13.5, 95CI: 4.42–41.18, p = 0.000005; PFS: 3.26, 95CI: 1.56–6.81, p = 0.002). A high D_mvcc_ per-10 µm was associated with a significantly increased risk of cancer-related death and progression by 98% (p = 0.0001) and 30% (p = 0.044), respectively.

**Conclusion:**

The NSCLC tissues had varying distances from microvessels to cancer cells, and long distances were strongly associated with poor survival.

## Introduction

Lung cancer is common and has high incidence and mortality globally (1). Non-small cell lung cancer (NSCLC) accounts for 80–90% of all lung cancers. Angiogenesis contributes to lung cancer development by supplying oxygen and nutrients and driving growth and metastasis (2). Accordingly, numerous factors related to angiogenesis-related molecules and morphological parameters have been exploited as potential prognostic factors for NSCLC. The prominent examples of architectural factors are microvessel area (MVA) and microvessel density (MVD). These two quantitative markers of the microvessels in tumor tissues have been the focus of research (3–5). However, their role in survival prediction remains controversial (6–9). One explanation for this discrepancy is that several vessels were separated from cancer cells by intratumoral stroma. Therefore, cancer cells may not receive adequate oxygen and nutrients if they lie too far from vessels, even when vascularization is dense (10–12).

In several solid tumors, the stroma is desmoplastic and filled with a thick extracellular matrix, including collagen, fibronectin, and laminins (9, 13). The stroma separates cancer cells from the vasculature (14–16). Before diffusion into cancer cells, oxygen, nutrition, and antitumor drugs should cross the stiff stroma and overcome the hamper by increasing interstitial fluid pressure and lengthening transport distance (9, 17, 18). The inadequate supply of oxygen and antineoplastic agents may induce hypoxia and chemoresistance, ultimately driving tumor progression (19–22). Therefore, we hypothesized that the distance between nutrition and drug transport is a promising factor for predicting the prognosis of NSCLC. However, the diffusion distance in tumor tissues has not been measured, and its correlation with survival outcomes also needs to be evaluated. In the present study, we measured the distance between microvessels and cancer cells (D_mvcc_) and evaluated its role in predicting the prognosis of patients with NSCLC.

## Materials and Methods

### Patients and Samples

This retrospective study reviewed NSCLC patients treated at the Zhejiang Cancer Hospital (Hangzhou, China) from July 2011 to October 2012. The patients included (1) were histologically diagnosed with primary NSCLC, (2) underwent surgical resection as primary treatment, (3) had available biopsied tissue of the primary lesion collected before chemotherapy, and had their full information, including clinicopathologic characteristics and survival outcomes, available. The data on the follow-up was updated on July 20th, 2016. Thoracic and abdominal CT, abdominal ultrasonography, MRI, and chest radiography were used to monitor tumor recurrence. Tumor tissues were obtained from the tissue bank of the Zhejiang Cancer Hospital. This study was approved by the Ethics Committee of Zhejiang Cancer Hospital (No. IRB-2017-67), and it adhered to the ethical principles of the Declaration of Helsinki. Written informed consent was obtained from all the patients.

### Immunohistochemical Staining

One specimen of resected lung cancer tumor tissue from each individual was collected. Microvessels were detected by immunohistochemical (IHC) staining of vascular endothelial cells for the CD31 antigen (Ca# 13063, Wuhan Goodbio Technology Co., Ltd) of vascular endothelial cells. In brief, sections of paraffin-embedded tumor tissues (5 μm thick) were de-paraffinized and rehydrated. The deparaffinized sections were incubated with the primary antibody (rabbit polyclonal antibody, Proteintech, Rosemont, USA) at a dilution of 1:300 overnight at 4°C after pretreatment with Dako EnVisionTM FLEX Target Retrieval Solution (high pH, pH 9.0, Ca# K5007, Dako) at 95°C for 20 min for antigen retrieval. The sections were stained with secondary antibodies for 30 min at room temperature. Next, the color was developed with 3,3’-diaminobenzidine in Tris–HCl (50 mmol/L, pH 7.5) containing 0.005% hydrogen peroxide, followed by counterstaining with hematoxylin.

### Measurement of D_mvcc_

D_mvcc_ was defined as the distance from each microvessel to its nearest neighbor cancer cell.

### Microvessels and “Hot Spot”

The image analysis procedure is illustrated in **Figure S1**. Microvessels were identified based on specific architectures; the lumen lined by endothelial cells was positively visualized with anti-CD31 staining. Microvessels in the tumor tissue were observed with a fluorescence microscope (Nikon Eclipse TI-SR) equipped with a Nikon DS-U3 digital camera controller. After an overview of the section, the field with the highest density of microvessels was selected as the “hot-spot” field of each section according to a previously reported method (23).

### Distance Measure

The distances between each tumor microvessel and its near cancer cells were dependently measured at 200× magnification by two experienced investigators using the Image J software (Wayne Rasband, National Institute of Health, USA), and the shortest distance was identified as the D_mvcc_ of individual tumor vessel. Subsequently, the mean D_mvcc_ of all vessels in the “hot spot” field of individual patients was calculated as the patient’s D_mvcc_.

### Statistical Analyses

Statistical analysis was performed using SPSS Statistics (Version 23.0, IBM Inc., New York, USA), R Studio software (Version 0.99.486, R Studio, Inc.), and Prism 7 (GraphPad Software Inc., La Jolla, CA, USA). Where appropriate, linear regression analysis (Pearson correlation coefficient), analysis of variance (ANOVA), and Student’s t-tests were used. Overall survival (OS) and progression-free survival (PFS) were defined as the interval between the first diagnosis and death or the first evidence of disease progression, respectively. The Kaplan–Meier method was used for the univariate analysis of OS and PFS, and they were compared using the log-rank test. The discriminative performance of the prognostic survival model was evaluated using the concordance index (C-index). To determine the optimal cutoff value of D_mvcc_ for OS and PFS prediction, values from 2–50 µm (step size = 1 µm) were taken as 49 potential cutoff values. The patients were stratified into the long or short D_mvcc_ group according to one of the 49 cutoff values, and the HRs of OS and PFS were calculated using the log-rank test. The cutoff value with the most significant correlation between OS and PFS was defined as the optimal cutoff value. The correlation between D_mvcc_ and survival outcome (OS or PFS) was examined using various categorical strategies of D_mvcc_: (1) dichotomy by median value; (2) dichotomy by optimal cutoff; (3) trichotomy: bottom tertile, middle tertile, and top tertile; (4) quartation: 1st, 2nd, 3rd, and 4th quartile; (5) per-10 µm increase: 0–10 µm, 10–20 µm, 20–30 µm, 30–40 µm, 40–50 µm. A stepwise multivariate Cox proportional hazards regression was performed to further test for the independence of D_mvcc_ measurements from potential prognostic variables (such as age, sex, smoking history, tumor histology, tumor differentiation, disease stage, or chemotherapy). A series of predetermined subgroup analyses was conducted to evaluate the prognostic value of D_mvcc_ in sub-populations with various clinical pathologies, including age, sex, smoking history, tumor histology, age, disease stage, tumor differentiation, or chemotherapy. All tests were two-sided, and statistical differences were considered significant at *p <*0.05.

## Results

### Patients

A total of 100 patients (74 men) with 54 adenocarcinomas and 43 squamous cell carcinomas were included. The age of the patients ranged from 40 to 79 years, with a median age of 59 years. The TNM stage was I/II and III/IV in 71 and 29 subjects, respectively. A total of 1, 47, and 49% of the patients had well-, moderately, and poorly differentiated tumors, respectively. During the follow-up period (median, 51.1m; range, 45.5 to 60.0m), 29 patients died (29%) and 44 experienced recurrence (recurrence outcome was unavailable for eight subjects). Of the 81 patients who received chemotherapy, 83% received single-line platinum-based regimens: GP regimen (45 patients, gemcitabine plus cisplatin), DP regimen (nine patients, docetaxel and cisplatin), GC regimen (four patients, gemcitabine plus cisplatin), NP regimen (four patients, vinorelbine plus cisplatin), and PC regimen (five patients, paclitaxel plus carboplatin).

### D_mvcc_

**Figure 1:**
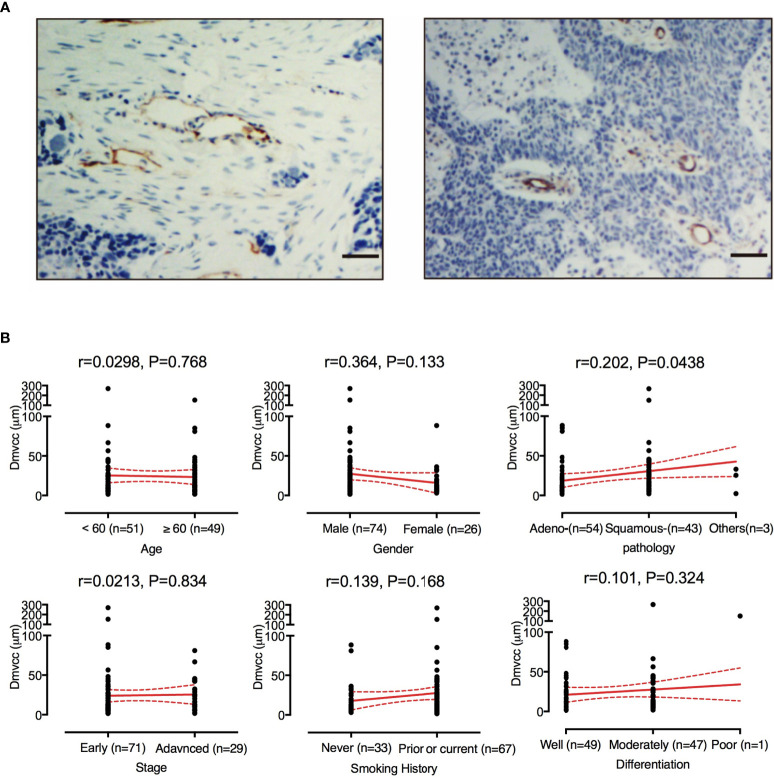
Evaluation of D_mvcc_ in NSCLC tissue sections. **(A)** Immunostaining of endothelial cells for CD31 (brown) with long D_mvcc_ (left panel) and short D_mvcc_ (right panel) in NSCLC tissue sections. Bar: 50 μm, **(B)** Correlates of D_mvcc_ associated with age, gender, smoking history, tumor histology, tumor differentiation, stage, and chemotherapy regimens. The mean value (red full-line) and its 95%CI (red dotted line) are shown.

The microvessels were visualized by IHC staining of the CD31 antigen. As shown in **Figure 1A**, the microvessel was surrounded by tumor stroma and away from the cancer cells. A total of 32–569 vessels (mean: 112 ± 77; median: 91) per patient were investigated. The individual D_mvcc_ ranged from 1.61 to 269.70 μm (mean value of 24.37 μm ± 33.45 μm and 1st, 2nd, and 3rd quartile values of 7.34, 13.15, and 30.76 μm). Most of the patients (93 of 100) had a mean D_mvcc_ of less than 50 μm. In all the participants, we recorded a significant correlation between D_mvcc_ and the pathological type (*r* = 0.202, *p* = 0.044), and a longer D_mvcc_ was observed in the tissues of squamous carcinoma than in those of adenocarcinoma (33.7 μm ± 44.6 μm *vs.* 17.2 μm ± 19.6 μm, **Figure 1B**). However, no significant correlations with the other traits were detected.

### Correlation of D_mvcc_ and Survival Outcome

**Figure 2:**
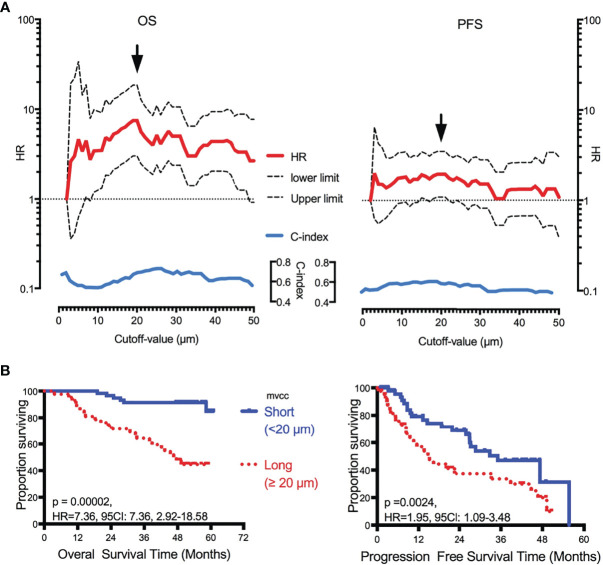
Correlation of D_mvcc_ and survival outcome. **(A)** Significant test: the hazard ratios (HRs, red full-line, long- *vs* short-D_mvcc_) of OS (right panel) and PFS (left panel) for various cutoff values of D_mvcc_ (from 2 to 50 μm at a step of 1 μm, bottom of the figures) were demonstrated with its upper limit (upper dotted-line) and lower limit (lower dotted-line) of 95% CI. Additionally, the c-index values were displayed as blue full-line. The optimal cutoff-value was demonstrated by a solid arrow. **(B)** Prognostic significance of D_mvcc_ in NSCLC patients. Kaplan–Meier survival curves show OS (left panels) and PFS (right panels) for patients presenting a long (full line) or short (dotted line) D_mvcc_ dichotomized by the optimal cutoff-value (20 μm, **B**).

Of the 49 candidate cutoff values (from 2 to 50 μm, step = 1 μm), 41 (83.7%, 7 μm, and 9–48 μm), and seven (14.3%, 16–21 μm, and 24 μm) played significant roles in predicting OS and PFS, respectively (**Figure 2A**). A D_mvcc_ of 20 μm was defined as the optimal cutoff value for both OS (HR = 7.36, 95CI = 2.92–18.58, *p* = 0.00002) and PFS (HR = 1.95, 95CI = 1.09–3.48, *p* = 0.00006) (**Figure 2B**).

**Figure 3:**
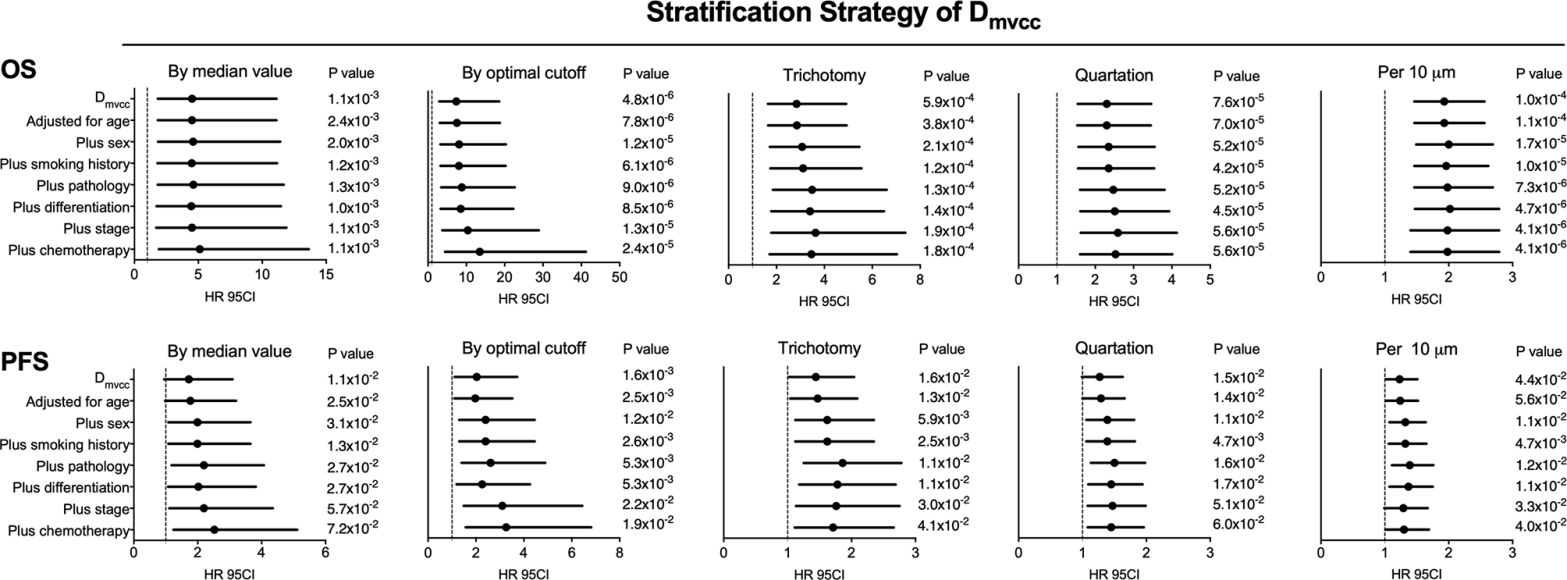
Adjusted correlation of D_mvcc_ and survival outcome. The hazard ratios (HRs) of OS (upper panel) and PFS (lower panel) for various categorical strategies of D_mvcc_: (1) dichotomy by median value; (2) dichotomy by optimal cutoff; (3) trichotomy: bottom tertile, middle tertile, and top tertile; (4) quartation: 1st, 2nd, 3rd, and 4th quartiles; (5) per-10 µm increase: 0–10, 10–20, 20–30, 30–40, and 40–50 µm after successively adjusting by age, sex, smoking history, tumor histology, tumor differentiation, disease stage, and chemotherapy.

To determine the prognostic role of D_mvcc_, five sets of categorical continuous D_mvcc_, such as median-value dichotomy, optimal cutoff value dichotomy, tertiles, quartile, and per-10-µm increase, were tested. Poor survival outcomes (both OS and PFS) were noted in patients with long D_mvcc_ than in those with short D_mvcc_ based on any of the five stratification conditions (OS: HR = 1.93–7.36, *p* = 0.001–0.00004; PFS: HR = 1.23–1.95, *p* = 0.041–0.024) (**Table 1**). In analyses based on adjustments for age, sex, smoking history, pathology, differentiation, stage, and chemotherapy, the long D_mvcc_ predicted poor survival with median HRs of 1.98–13.50 for OS and 1.30–3.26 for PFS based on various categorical strategies (**Figure 3**). After progressive adjustment for various risk factors, D_mvcc_ remained significantly associated with NSCLC-related death or progression. For a per-10 µm increase in D_mvcc_, the HRs for NSCLC-related death and progression were 1.98 (95CI: −1.40–2.79) and 1.30 (95CI: −1.01–1.69) in the final multivariable model (**Figure 3**). The median PFS was significantly shortened from 35.1 months (95%CI: 22–1-48.1 months) for short D_mvcc_ (<20 µm) patients to 15.1 months (95%CI: 8.6–21.6 months) for long D_mvcc_ (≥20 µm) patients (*p* = 0.024, **Figure 2B**). As disclosed in the predetermined subgroup analysis, D_mvcc_ was a promising prognostic marker of OS for older ages (age ≥60 years), younger ages (age <60 years), male sex, squamous carcinoma, adenocarcinoma, poor tumor differentiation, advanced stage, early stage, smoking or prior smoking, and prior chemotherapy, as well as a promising prognostic marker of PFS in patients who are male, have squamous carcinoma, are current or former smokers, and have had chemotherapy (**Figure S2**).

**Table 1:** Association of D_mvcc_ with survival outcome using various categorical strategies.

Categorical D_mvcc_	OS			PFS		
	HR, 95CI	P-value	C-index	HR, 95CI	P-value	C-index
Dichotomy by median value (13 µm)^*^	4.51, 1.83–11.12	0.001	0.67	1.72, 0.95–3.09	0.072	0.59
Dichotomy by optimal cutoff (20 µm)^*^	7.36, 2.92–18.58	0.00002	0.74	1.95, 1.09–3.48	0.024	0.61
Trichotomy	2.84, 1.64–4.91	0.0002	0.72	1.44, 1.02–2.04	0.041	0.61
Bottom tertile	1			1		
Middle tertile	1.59, 0.47–5.44	0.459		1.45, 0.68–3.10	0.334	
Top tertile	6.32, 2.13–18.78	0.0009		2.07, 1.03–4.18	0.042	
Quartation	2.30, 1.54–3.46	0.00006	0.74	1.27, 0.99–1.63	0.060	0.61
1st quartile	1			1		
2nd quartile	0.93, 0.19–4.65	0.932		0.91, 0.36–2.25	0.830	
3rd quartile	2.91, 0.77–10.98	0.115		1.85, 0.83–4.15	0.134	
4th quartile	7.95, 2.28–27.74	0.001		1.81, 0.83–3.95	0.137	
Per-10 µm increase	1.93, 1.46–2.56	0.000004	0.76	1.23, 1.01–1.51	0.040	0.63
0–10 µm	1			1		
10–20 µm	0.73, 0.13–3.99	0.715		1.25, 0.52–3.01	0.624	
20–30 µm	4.86, 1.46–16.18	0.010		2.24, 0.998–5.02	0.051	
30–40 µm	7.12, 1.76–28.82	0.006		2.53, 0.80–8.04	0.114	
40–50 µm	10.69, 3.08–37.15	0.0002		2.00, 0.80–5.03	0.140	

^*^The lower as 1.

### Long D_mvcc_ Independently Indicated Poor Prognosis

Several multivariate analyses (**Table 2**) of features, including sex, smoking history, tumor histology, age, disease stage, tumor differentiation, D_mvcc_, and chemotherapy, were performed with five multivariable models based on different stratified strategies of D_mvcc_. D_mvcc_ was found to be an independent predictor of tumor-related death (*p* = 0.001–0.000005) or progression (*p* = 0.044–0.002) in all five models. The model based on the 10-μm-stratified D_mvcc_ was disclosed as the optimal model for survival prognosis, with the highest c-index values of 0.830 and 0.771 for OS (HR = 1.98, 95CI: 1.40–2.79, *p* = 0.0001) and PFS (HR = 1.30, 95CI: 1.01–1.69, p = 0.044), respectively. In addition, an advanced stage was suggested as another independent prognostic factor of OS (HR = 2.51–3.33, *p* = 0.027-0.008), but it showed no significant correlation with PFS (**Table 2**).

**Table 2:** Independent prognostic factors of NSCLC patients based on various multivariate analysis models.

Factors	Model 1 (dichotomy by median value)			Model 2 (dichotomy by optimal value)			Model 3 (Trichotomy)			Model 4 (Quartation)			Model 5 (Per 10 μm increase)		
	HR (95CI)	P value	C-index	HR (95CI)	P value	C-index	HR (95CI)	P value	C-index	HR (95CI)	P value	C-index	HR (95CI)	P value	C-index
OS															
D_mvcc_	5.11 (1.91–13.64)	0.001	0.790	13.5 (4.42–41.18)	0.000005	0.827	3.46 (1.71–7.03)	0.0006	0.792	2.53 (1.60–4.01)	0.0001	0.812	1.98 (1.40–2.79)	0.0001	0.830
Age	1.34 (0.62–2.93)	0.459		2.45 (1.00–6.02)	0.050		1.67 (0.72–3.90)	0.235		1.67 (0.73–3.78)	0.222		2.05 (0.77–5.42)	0.148	
Gender	2.64 (0.31–22.47)	0.375		7.32 (0.76–70.55)	0.085		4.89 (0.54–44.08)	0.157		4.08 (0.46–36.03)	0.206		–	0.935	
Smoking History	2.53 (0.31–20.61)	0.384		3.68 (0.44–30.6)	0.229		4.43 (0.54–36.73)	0.168		3.91 (0.47–32.31)	0.206		–	0.937	
Pathology	0.45 (0.16–1.23)	0.121		0.33 (0.11–1.02)	0.055		0.43 (0.15–1.23)	0.114		0.41 (0.14–1.18)	0.098		0.51 (0.17–1.58)	0.243	
Differentiation	1.06 (0.47–2.39)	0.887		1.08 (0.46–2.56)	0.856		0.96 (0.43–2.15)	0.912		0.99 (0.43–2.27)	0.980		1.16 (0.46–2.92)	0.756	
TNM Stage	2.51 (1.11–5.68)	0.027		3.33 (1.38–8.04)	0.008		2.92 (1.24–6.85)	0.014		2.85 (1.23–6.57)	0.014		3.32 (1.27–8.67)	0.014	
Chemotherapy	3.64 (0.72–18.54)	0.120		4.93 (0.84–28.93)	0.077		1.76 (0.35–8.96)	0.495		2.30 (0.45–11.75)	0.312		1.90 (0.34–10.51)	0.462	
PFS															
D_mvcc_	2.52 (1.24–5.11)	0.011	0.763	3.26 (1.56–6.81)	0.002	0.772	1.71 (1.11–2.66)	0.016	0.763	1.45 (1.08–1.96)	0.015	0.762	1.30 (1.01–1.69)	0.044	0.771
Age	0.82 (0.43–1.56)	0.540		1.09 (0.54–2.19)	0.815		0.86 (0.44–1.70)	0.672		0.87 (0.45–1.70)	0.691		0.97 (0.48–1.97)	0.934	
Gender	2.75 (0.75–10.13)	0.127		3.95 (1.01–15.47)	0.049		2.83 (0.76–10.60)	0.121		2.80 (0.75–10.48)	0.126		3.11 (0.67–14.45)	0.147	
Smoking History	1.72 (0.48–6.20)	0.410		1.78 (0.49–6.44)	0.376		2.16 (0.59–7.84)	0.243		1.90 (0.52–6.87)	0.329		1.91 (0.41–8.84)	0.407	
Pathology	0.39 (0.16–1.00)	0.050		0.44 (0.18–1.10)	0.078		0.39 (0.15–1.01)	0.052		0.40 (0.15–1.02)	0.054		0.45 (0.17–1.22)	0.118	
Differentiation	0.60 (0.30–1.18)	0.139		0.59 (0.30–1.16)	0.126		0.57 (0.29–1.12)	0.102		0.58 (0.29–1.14)	0.116		0.60 (0.29–1.23)	0.162	
Stage	2.86 (1.48–5.51)	0.002		3.56 (1.76–7.20)	0.000		2.82 (1.45–5.49)	0.002		2.92 (1.50–5.65)	0.002		2.96 (1.44–6.06)	0.003	
Chemotherapy	2.78 (0.92–8.43)	0.070		2.48 (0.84–7.32)	0.100		2.00 (0.68–5.85)	0.206		2.10 (0.72–6.13)	0.174		2.06 (0.69–6.20)	0.197	

## Discussion

Due to the role of the extracellular matrix in NSLC development, subjects with rich stroma were investigated for poor survival (24). Poor survival can be attributed to various profiles of stroma or alterations of rich stroma, such as the proportion of stroma, fibrosis extent (25), impaired vascularization (26, 27), and immune infiltration of the peritumoral stroma (28) and the evidence related to the aforementioned contributors has been explored. For instance, squamous cell carcinoma of patients with fibrous stroma demonstrated a more invasive phenotype and was associated with a significantly poor prognosis (25). However, the impact of the elongated distance of diffusion on survival outcomes has not been focused on. Therefore, the correlation between perfusion distance and patient survival was evaluated in the present study. As expected, a long D_mvcc_ was predictive of poor survival. This suggests that the long distances from vessels to cancer cells partly affected patient survival.

A short diffusion distance between cancer cells and microvessels is crucial for efficient nutrient supply. Enlarged perfusion paths may induce an insufficient supply of oxygen, nutrition, and anti-tumor drugs, ultimately improving tumor growth (29, 30). However, the diffusion distance in the tumor tissues of patients and their correlation with survival outcomes have seldom been reported. Therefore, in the present study, we developed a method to evaluate the distance of nutrient diffusion and examine its role in prognostic prediction. Because the concept of the “distance between cancer cells and vessels” is seldom reported, no definition and measurement methodology can be used. We defined D_mvcc_ as the distance between each microvessel and its nearest cancer cell for the following two reasons. First, the vessel was the center of the blood perfusion area; thus, the distance was measured based on each vessel; moreover, the nearest distance was easy to measure instead of the median or farthest distance. As we disclosed, the mean D_mvcc_ value of most patients ranged from 2 to 50 μm, and the mean D_mvcc_ in half of the patients was <13 μm. It was really interested that longer D_mvcc_ was found in the tissues of squamous carcinoma than adenocarcinoma. It may be attributed to distinct tumor-accociated fibroblasts (TAFs), the cell built extra-cellular matrix, between squamous carcinoma and adenocarcinom. It was reported that TAFs in squamous carcinoma exhibited higher levels of matrix rigidity related factors, such as FAK, β1 expression, and ERK1/2 than TAFs in adenocarcinom. Matrix stiffening induced a larger TAF accumulation in squamous carcinoma compared with adenocarcinom. Therefore, a larger proportion of matrix caused longer distance were observed in squamous carcinoma (31).

The optimal cutoff value of D_mvcc_ was determined by a test that included 49 candidate values (2–50 μm, step = 1 μm). As a result, 20 μm was determined to be the optimal cutoff value. There were 58 patients with short D_mvcc_ (<20 μm) and 42 patients with long D_mvcc_ (≥20 μm). Five categorical strategies, such as per-10μm increase, quartation, trichotomy, dichotomy by median value (13 µm), and dichotomy by optimal cutoff (20 µm), were used to evaluate the role of the D_mvcc_ in survival prediction. A long D_mvcc_ significantly predicted shorter OS of patients based on all five categorical strategies. A significantly shorter PFS was observed in patients with long D_mvcc_ than in those with short D_mvcc_ (<20 µm, or per-10 µm decreased). As disclosed, the risks of cancer-related death and progression were increased by 93% (HR = 1.93, 95CI: 1.46–2.56, *p* = 0.000004) and 23% (HR = 1.23, 95CI: 1.01–1.51, *p* = 0.040), respectively, per 10 µm increase in D_mvcc_. Moreover, D_mvcc_ and stage were also proven to be independent prognostic factors based on five multivariate analysis models. The models that were based on D_mvcc_ dichotomized by the optimal value (c-index: 0.827 of OS and 0.772 of PFS) and every 10 μm increase (c-index: 0.830 of OS and 0.771 of PFS), respectively, were recommended as the best two prognostic prediction models.

These results can be attributed to the impaired distribution of oxygen, nutrition, and anticancer drugs hampered by prolonged drug penetration from blood vessels to cancer cells. In tumor tissue, microvessels are separated from cancer cells by abnormally dense stroma, which consists of high levels of collagenous fibers and stabilized polysaccharide networks (hyaluronate and proteoglycans) (20). Above all, a long D_mvcc_ indicates poor blood-supply efficiency. The diffusion of oxygen was inversely proportional to the square value of the perfusion distance *in silicon* (32). *In vivo*, at a distance of 50 μm from the vessel, the oxygen partial pressure (pO_2_) was decreased by approximately 40 and 50% in xenografts of breast cancer and NSCLC cancer (33, 34). In addition, pO_2_ decreased by 100% at a distance of 70 μm from the vessels in breast cancer xenografts (35). The diffusion of glucose also decreased by approximately 40% at a diffusion distance of 100 μm (33). The hypoxic microenvironment is widely accepted as a driver of tumor growth and the cause of therapy resistance (36–39).

The mobility of drugs in penetrating the extracellular matrix is also limited by cell–cell adhesion, high interstitial fluid pressure, lack of convection, drug metabolism, and binding (9, 40). Recent data suggest that inefficient delivery of antineoplastic drugs in the tumor environment is a novel and important contributor to chemoresistance (19, 20, 41–43). A prolonged distance from blood vessels to cancer cells is a significantly difficult route for antitumor agent delivery. This led to a steep decrease in the drug concentration around cancer cells. As reported, the intensity of doxorubicin decreased to half from the nearest blood vessel at a distance of 40–50 μm (44). The incomplete intratumoral distribution of gemcitabine and fluorouracil, which induced the reverse impact of the antitumor effect, has been addressed *in vivo* (21, 45). Our study showed a significant association between a long D_mvcc_ and poor survival outcomes of chemotherapy in patients with NSCLC. It has been suggested that D_mvcc_ is a surrogate biomarker for the prediction of survival outcomes in NSCLC patients.

There are several limitations to the present study that should be addressed. First, perfusion distance was the only contributor to poor outcomes; other factors, such as fibrosis extent and impaired microvessel pattern, should be evaluated. Moreover, only intratumor vessels in the field of “hot spots” were limited to capacity. This may have led to sampling bias. As a novel characteristic of the perfusion system, the definition and measurement of D_mvcc_ need to be validated. At last, it was expected that short distance between cancer and blood vessels, would facilitate cancer cells accessing to vessels and improve the chance of metastasis and cause worse patience survival. However, the correlation between D_mvcc_ and metastatic tumor was not estimated in the present study.

In conclusion, the present study proves that a long distance from an intratumor microvessel to cancer cells is an independent predictive factor of poor survival outcomes in NSCLC patients. It provides clinical insights into the chemoresistance caused by the long penetration distance-induced impaired accumulation of antineoplastic agents in tumors.

## Data Availability Statement

The original contributions presented in the study are included in the article/**Supplementary Material**. Further inquiries can be directed to the corresponding authors.

## Ethics Statement

The studies involving human participants were reviewed and approved by the Ethics Committee of Zhejiang Cancer Hospital. The patients/participants provided their written informed consent to participate in this study. Written informed consent was obtained from the individual(s) for the publication of any potentially identifiable images or data included in this article.

## Author Contributions

HD, LF, and YT contributed to the conception and design of the study. JS, WX and LZ carried out the experiments. JS, YT, YC, and JZ revised the manuscript. YC, YS, YZ and LZ contributed to data collection and analysis. All authors contributed to the article and approved the submitted version.

## Funding

This work was supported by the National Natural Science Foundation of China (81773819, 81973396 and 82003851), Natural Science Foundation of Zhejiang Province (No: Q17H300007), Natural Science Foundation of Zhejiang Province (LY19H160006), Science and Technology in Zhejiang Province Chinese Medicine Program (No: 2015ZA036, and 2019KY473), Scientific Research key program of Health Bureau of Zhejiang Province (WKJ-ZJ-1504), Medical Science Research Foundation of Zhejiang Province (No: 2015ZDA006), Zhejiang Provincial Program for 151 Talents (LF), and Zhejiang Cancer Hospital Program for the Cultivation of 1022 Talents (LF).

## Conflict of Interest

The authors declare that the research was conducted in the absence of any commercial or financial relationships that could be construed as a potential conflict of interest.
