# Supplementary figures and images for: Long Distance From Microvessel to Cancer Cell Predicts Poor Prognosis in Non-Small Cell Lung Cancer Patients
Source: Front Oncol. 2021 Jun 11;11:632352. doi: 10.3389/fonc.2021.632352 (PMC8226084; doi:10.3389/fonc.2021.632352)

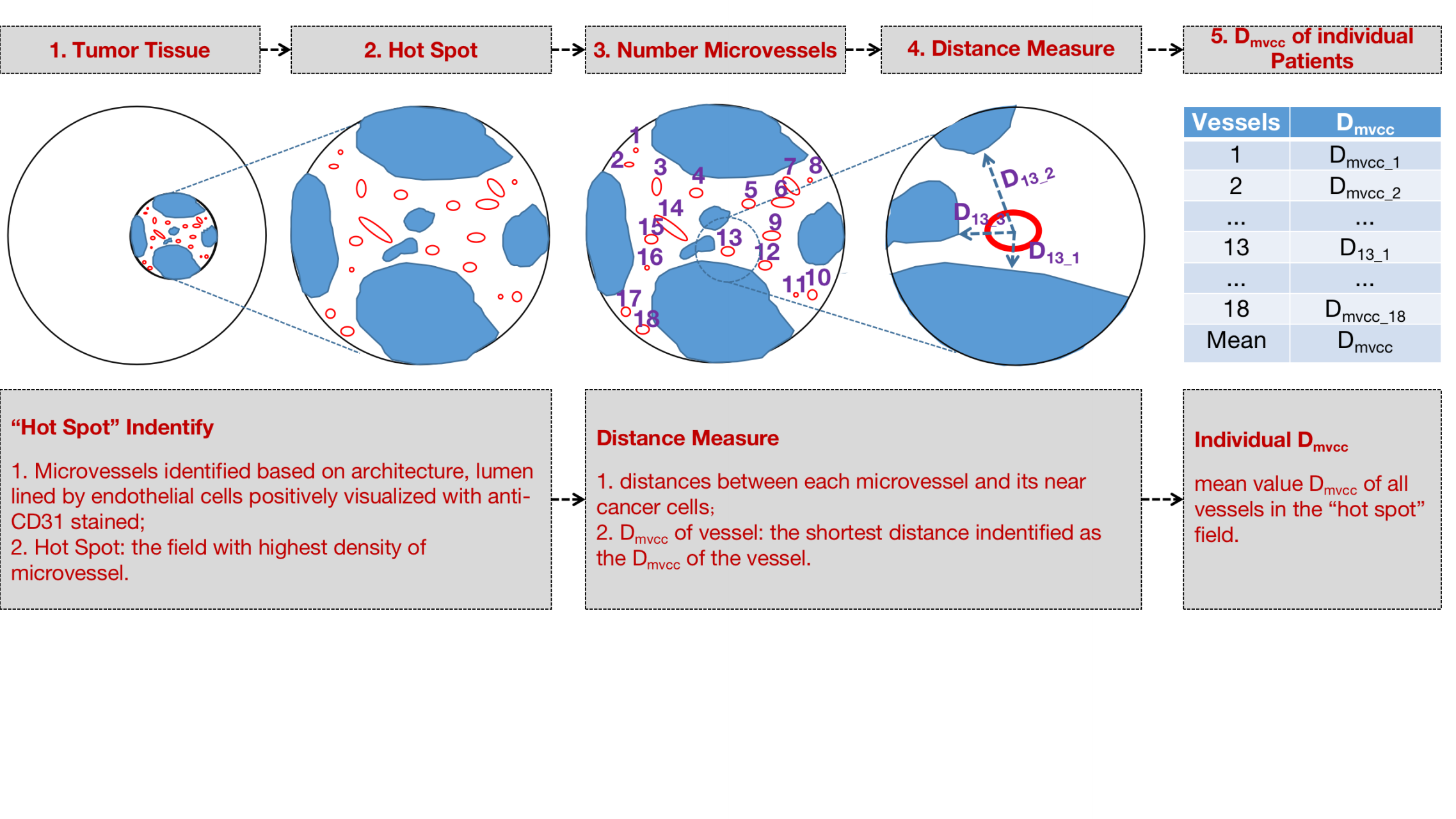

Supplement: Supplementary file 1 [file Image_1.tif]

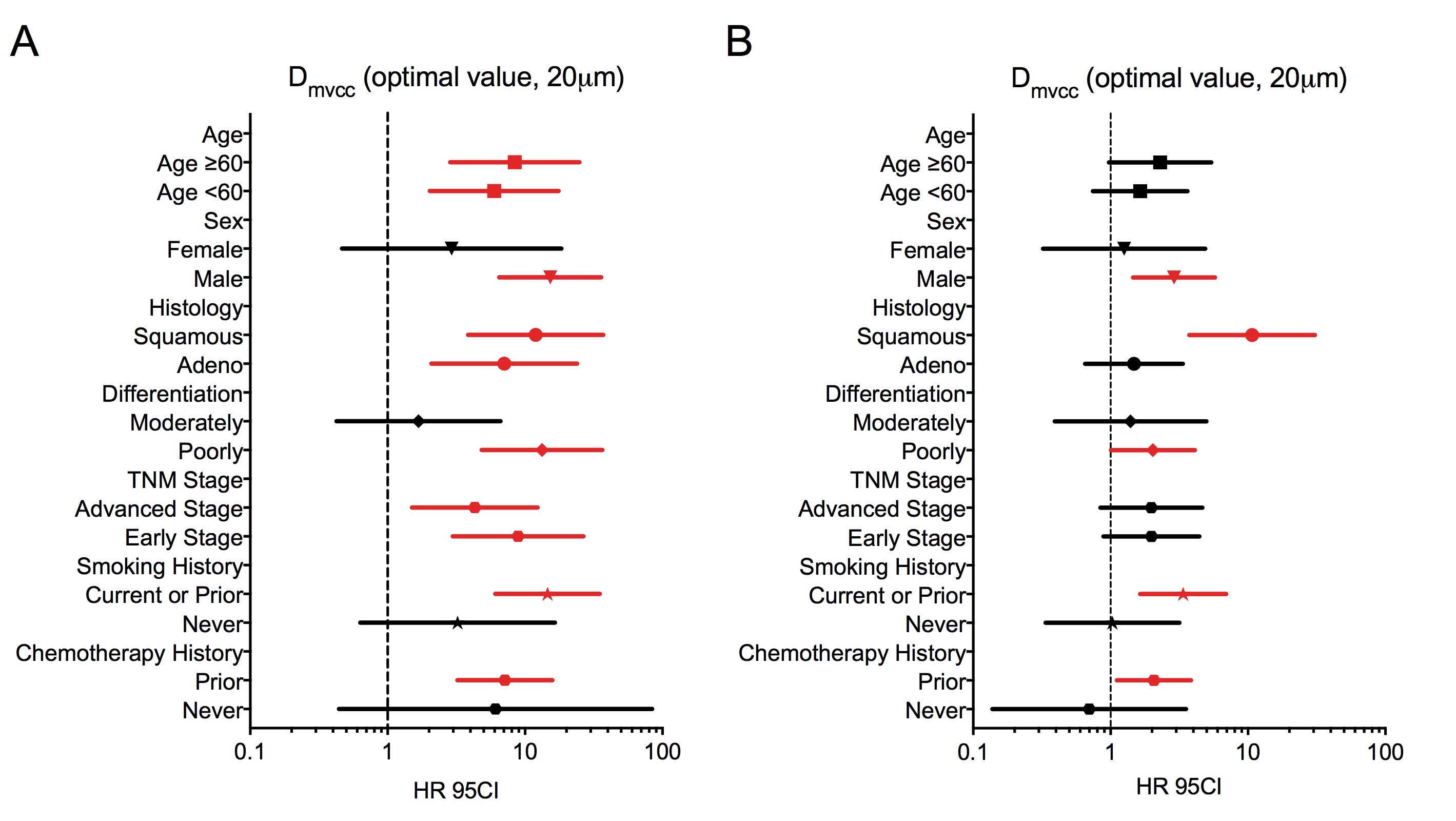

Supplement: Supplementary file 2 [file Image_2.tiff]
